# A Dual Filter Based on Radial Basis Function Neural Networks and Kalman Filters with Application to Numerical Wave Prediction Models

**DOI:** 10.3390/s24248006

**Published:** 2024-12-15

**Authors:** Athanasios Donas, Ioannis Kordatos, Alex Alexandridis, George Galanis, Ioannis Th. Famelis

**Affiliations:** 1 Department of Electrical and Electronic Engineering, University of West Attica, Ancient Olive Grove Campus, 250, Thivon Ave., Egaleo, 12241 Athens, Greece; adonas@uniwa.gr (A.D.); ikordatos@uniwa.gr (I.K.); ifamelis@uniwa.gr (I.T.F.); 2 Hellenic Naval Academy, Hatzikiriakion, 18539 Piraeus, Greece; ggalanis@hna.gr

**Keywords:** Kalman filters, post-process algorithms, radial basis function neural networks, significant wave height, WAM

## Abstract

The aim of this study is to introduce and evaluate a dual filter that combines Radial Basis Function neural networks and Kalman filters to enhance the accuracy of numerical wave prediction models. Unlike the existing methods, which focus solely on systematic errors, the proposed framework concurrently targets both systematic and non-systematic parts of forecast errors, significantly reducing the bias and variability in significant wave height predictions. The produced filter is self-adaptive, identifying optimal Radial Basis Function network configurations through an automated process involving various network parameters tuning. The produced computational system is assessed using a time-window procedure applied across divergent time periods and regions in the Aegean Sea and the Pacific Ocean. The results reveal a consistent performance, outperforming classic Kalman filters with an average reduction of 53% in bias and 28% in RMSE, underlining the dual filter’s potential as a robust post-processing tool for environmental simulations.

## 1. Introduction

Accurate wave predictions have become increasingly important in recent decades due to several affected activities, such as research and rescue, tourism, shipping, and renewable energy sources. Numerical Wave Prediction (NWP) models, which are gradually being employed by operational centers to successfully mimic environmental conditions on a worldwide scale, are a dependable and efficient way to accomplish these goals.

However, when forecasting wave parameters in a specific geographic region of interest, NWP models often struggle to give adequate results. This is due to the complex interplay between multiple factors, including the strong reliance on initial and lateral conditions, the challenge of capturing small-scale phenomena, and the parametrization of certain wave processes [1].

To avoid such issues, one feasible option would be to increase the NWP model’s resolution; however, the efficiency of this methodology is unknown, and the computational cost will surely increase dramatically. A different strategy would be to utilize post-processing algorithms to improve the direct output of the NWP model in use or to employ assimilation systems to enhance its initial conditions. Galanis et al. [2] introduced a strategy that enhances the effect of data assimilation on predicting ocean waves, demonstrating improved accuracy via integrated modeling techniques. Famelis et al. [3] investigated both classical and Quasi-Newton methods to optimize the prediction of meteorological parameters, while Famelis and Tsitouras [4] proposed a quadratic shooting solution for environmental parameter prediction, which effectively addresses complex boundary conditions.

Building on these foundational advancements, Dong et al. [5] developed a hybrid data assimilation system incorporating machine learning to augment numerical weather prediction models, addressing limitations inherent in traditional methods. Similarly, Rojas-Campos et al. [6] applied deep learning techniques to post-process NWP precipitation forecasts, significantly improving the predictive accuracy. Furthermore, Krasnopolsky [7] conducted a comprehensive review of machine learning applications in data assimilation and model physics, emphasizing the transformative potential of these technologies.

Finally, recently, Kordatos et al. [8] further explored the application of Radial Basis Function neural networks for predicting significant wave height, demonstrating their efficacy in improving forecasts through spatially nested datasets. Collectively, these studies illustrate the critical role that advanced numerical methods and machine learning play in enhancing the accuracy and reliability of environmental predictions, with broad implications for sectors such as marine operations and climate research.

The proposed methodology is among the post-processing algorithms. More precisely, it aims to improve the predictions of an NWP model by reducing the systematic and non-systematic parts of the simulation error. Systematic errors, also known as biases, are consistent and predictable deviations caused by inherent deficiencies in the model, such as flawed parameterizations or incomplete representation of physical processes. These errors persist over time or specific conditions, making them identifiable and correctable through techniques like bias correction or model calibration.

On the other hand, non-systematic errors are random and unpredictable deviations arising from factors such as incomplete observations, numerical noise, or unresolved small-scale phenomena (wave shoaling, wave refraction, diffraction, etc.). Their lack of a consistent pattern makes them more challenging to mitigate, underscoring the chaotic and stochastic nature of the simulated system. Addressing both types of errors is crucial for enhancing the accuracy and reliability of environmental predictions.

The first objective has been extensively discussed by several researchers, who have developed various tools to address it, like ANN mechanisms [9,10] or sophisticated statistical models [11,12,13]. In our approach, however, we utilize the Kalman filter (KF) algorithm to remove such errors [14,15,16]. The Kalman filter is considered the fastest sequential approach [17] that combines recent forecasts with recursively observed data. Thus, its low CPU memory demand provides a significant benefit for every application.

In many cases, though, KFs are unable to detect and, hence, decrease the non-systematic part of the forecast error [18], resulting in poor and unstable final predictions. To tackle this challenge, a Radial Basis Function neural network (RBF nn) is applied in this work, acting as an additional filter after Kalman’s initial implementation, with the goal of constraining the associated forecast uncertainty.

Under this framework, this study introduces a novel dual filter that uniquely combines Radial Basis Function neural networks with Kalman filters to enhance significant wave height forecasts obtained from the WAve Model (WAM). Unlike existing methodologies [19,20], the produced system is designed to simultaneously eliminate systematic biases and constrain the variability of the remaining non-systematic errors, resulting in more accurate and reliable final predictions. Moreover, another innovative aspect of the proposed system is its self-adaptiveness, which automatically determines the optimal RBF structure through hyperparameter optimization. This advanced capability ensures the robustness of the method across diverse regions and temporal scales, as illustrated via various case studies.

The suggested methodology was evaluated using an innovative time-window process application. Specifically, the former case study concerns the areas of Mykonos and Crete in the Aegean Sea for the years 2007–2009, while the latter case study concerns the region of 46002 in the Pacific Ocean for the years 2011–2013. In every case, the obtained results are compared to those derived from the standard Kalman filter to assess the efficacy of the suggested dual filter over classic methodologies.

The setup of the rest of the paper is: in Section 2, the main properties of the WAM model are described, along with a comprehensive analysis of the suggested methodology. Section 3 and Section 4 focus on the main elements of the Kalman filters and Radial Basis Function neural networks, while the time-window process application, together with the obtained results, is presented in Section 5. Finally, the extracted conclusions from the dual filter implementation are extensively discussed in Section 6.

## 2. Models and Methodology

This section describes the employed wave numerical model as well as a detailed analysis of the proposed methodology, emphasizing its key aspects.

### 2.1. Numerical WAve Model

The use of a well-established wave model is essential for the successful implementation of the proposed optimization strategy. For that reason, the third-generation numerical wave model WAM [21] was chosen, as it is frequently employed by a great number of operational and scientific organizations globally. WAM solves the wave transport equation (Equation (1)) directly using numerical schemes, without any assumption about specific shapes or types of the wave spectrum [22]:

(1) $$\frac{dF}{dt}+\frac{\partial }{\partial \phi }\left(\dot{\phi }F\right)+\frac{\partial }{\partial \Lambda }(\dot{\Lambda }F)+\frac{\partial }{\partial \theta }(\dot{\theta }F)=S,$$

where S is the source function, which includes white capping dissipation, wind forcing, and non-linear transfer. The variable F expresses the spectral density depending on frequencies (f), directions (θ), latitudes (φ), and longitudes (Λ).

More thoroughly, this work applies an adjustment of the WAM model, the improved version CY46R1 [23,24] of the ECMWF (European Centre for Medium Range Weather Forecasts). This enhanced version results in more accurate wave modeling and has been successfully implemented by many researchers [25,26,27], establishing a number of new and advanced parameterizations for spectral dissipation. Particularly, the enhancements include new extreme wave parameters based on the determination of the wave field’s kurtosis parameterizations [28], new parametrizations for the effect of shallow waters, and a new advection scheme that takes into consideration information from corner points [29].

The WAM model provides a lot of information about a variety of wave parameters, like the full wave spectrum at set grid points, the mean wave direction and frequency, the height and mean direction components of wind and swell waves, and wind stress fields that account for wave-induced stress and drag coefficient at each grid point at chosen output times. Nevertheless, this study emphasizes the Significant Wave Height (SWH), which is used in a range of modern applications (port electrification, etc.), and is defined as:

(2) $$SWH=4\sqrt{\int_{0}^{2\pi }\int_{0}^{\infty }f^{0}F\left(f,\theta \right)dfd\theta }.$$

### 2.2. Methodology

This study aims to develop a dual filter for numerical wave forecasts based on two widely used post-processing algorithms. Specifically, Kalman filters are sequentially combined with Radial Basis Function neural networks to improve significant wave height predictions from the WAM model. Initially, a non-linear Kalman filter process targets the systematic error of the simulation with the goal of producing the “corrected” data for the Radial Basis Function neural network implementation.

Afterward, the RBF network is trained for various combinations of activation functions, penalty parameters, and number of clusters to determine the optimal structure. More specifically, to determine the best RBF architecture, the “corrected” data are divided into the training and validation datasets. The former specifies the network’s weights based on the selected training algorithm, while the latter evaluates the model efficiency. The lowest validation error will resolve the optimal structure.

That process focuses on the remaining non-systematic part of the forecast bias, aiming to constrain its variability and the accompanying forecast uncertainty. That concludes the first phase of the proposed methodology, in which both the forecasts of the NWP model in use and the corresponding recorded observations are necessary. These data sets form the Training data set, which is utilized for the training process of the suggested dual filter.

When the training process is over, the optimum RBF topology is applied based on the independent Testing data set (forecasts & recorded observations) to generate improved forecasts for the wave parameter under study. The extracted outcomes are compared with the recorded observations and with those derived from a reference model, here the standard Kalman filter, to measure the degree of improvement offered by the proposed methodology.

**Figure 1:**
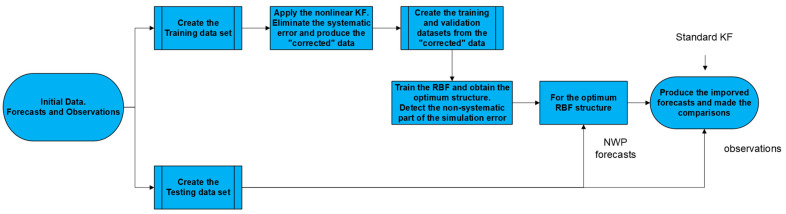
Method’s Diagram.

The key elements of the described method are presented in Figure 1.

**Figure 2:**
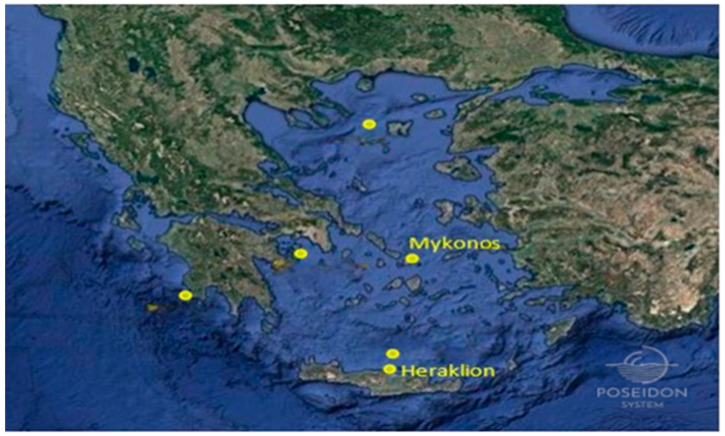
Locations of Aegean Stations (“https://poseidon.hcmr.gr/ (accessed on 11 December 2024)”).

**Figure 3:**
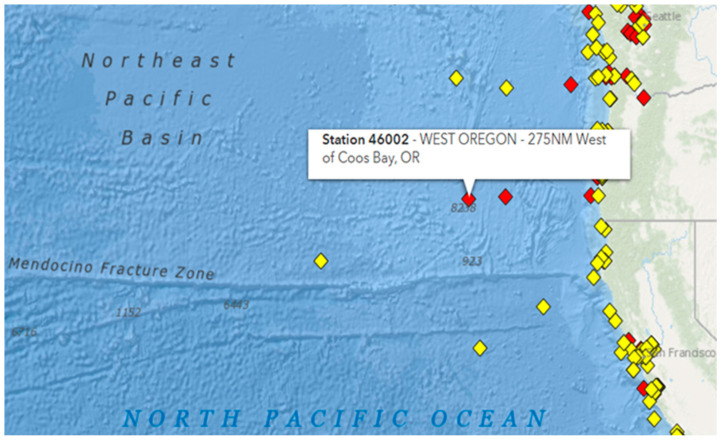
Location of Station 46002 (“https://www.ndbc.noaa.gov/ (accessed on 11 December 2024)”). Red squares indicate Stations with no data during the last 8 hours, while yellow squares indicate Stations with recent data.

As mentioned previously, the model’s predictions and the recorded observations are utilized by the dual filter. The predictions for the wave parameter are obtained from the NWP model, while the recorded observations are available from various stations in the Aegean Sea and in the Pacific Ocean. Specifically, the SWH observations from the Aegean Sea are derived from the Stations of Mykonos and Heraklion (Crete) in the area of Greece (Figure 2) and cover the period of 2007–2009. On the other hand, the corresponding observations from the Pacific Ocean are recorded by Station 46002 (Figure 3) and cover the period of 2011–2013.

The datasets from the Mykonos and Heraklion stations, as well as from station 46002, for the periods 2007–2009 and 2011–2013, were selected primarily due to the availability of high-quality, uninterrupted observations. Such data are essential for effectively training and validating the dual filtering methodology. Additionally, these periods exhibit significant variability in simulation errors, making them particularly suitable for evaluating whether the proposed optimization method enhances the predictive performance of the WAM model in areas where NWP models typically struggle. This selection is also appropriate to assess whether the suggested method overcomes the limitations of traditional forecasting approaches, such as the Kalman Filter, and delivers reliable final predictions.

## 3. Kalman Filters

Kalman filtering [30] is a set of mathematical formulations that compose a powerful and computationally efficient algorithm that estimates the evolution of an unknowing state vector x at time t, given information about a recorded vector y at the same time. It is assumed that the process of the state x from time t−1 to t is given by the following system equation:

(3) $$x_{t}=A_{t}*x_{t-1}+w_{t},$$

while the connection between $x_{t}$ and the observable vector $y_{t}$ is given by the measurement equation:

(4) $$y_{t}=B_{t}*x_{t}+v_{t}.$$

Combining Equations (3) and (4), the following state-measurement model is constructed:

$$x_{t}=A_{t}*x_{t-1}+w_{t}$$

$$y_{t}=B_{t}*x_{t}+v_{t}$$

where the variables $w_{t}$, $v_{t}$ are random vectors that follow the normal distribution with a zero mean, are independent, which means that *E*($w_{i}$·$v_{j}$) = 0 for any i,j ∈ N and also time-independent, which implies that *E*($w_{i}$·$w_{j}$) = 0 and $E(v_{i}\cdot v_{j})=0$, for all i≠j. The quantities $A_{t}$ and $B_{t}$ express the system and the measurement coefficient matrices, respectively, and need to be determined before the implementation of the filter.

After the state-space model is established, the Kalman filter algorithm applies the following steps:

- **Step 1**: Based on the vector $x_{t-1}$ and its error covariance matrix $P_{t-1}$, the optimal estimate for time t can be found by
  (5) $$x_{t/t-1}=A_{t}*x_{t-1}$$
  (6) $$P_{t/t-1}=A_{t}*P_{t-1}*{A_{t}}^{T}+W_{t}$$
- **Step 2**: When $y_{t}$ is available, the corrected value of $x_{t}$ at time t is calculated based on the following equations:
  (7) $$x_{t}=x_{t/t-1}+K_{t}*(y_{t}-B_{t}*x_{t/t-1}),$$
  where
  (8) $$K_{t}=P_{t/t-1}*{B_{t}}^{T}*{\left(B_{t}*P_{t/t-1}*{B_{t}}^{T}+V_{t}\right)}^{-1}$$
- **Step 3**: The new value of the covariance matrix of the unknown state $x_{t}$ is given by
  (9) $$P_{t}=(I-K_{t}*B_{t})*P_{t/t-1}$$

Equation (8) is known as the Kalman Gain, and it is the crucial parameter of the filter since it determines the way the filter will adjust to any possible new conditions [31]. For instance, a relatively small Kalman gain suggests high uncertainty in the measurements, meaning that only a small observation segment will be utilized for the new state prediction. Equations (5) and (6) present the prediction phase, while Equations (7) and (9) perform the correction phase. Finally, the parameters $W_{t}$ and $V_{t}$ are the covariance matrices of the random vectors $w_{t}$, $v_{t}$, respectively, also known as system and measurement noise covariance matrices.

To implement the Kalman filter’s algorithm, initial values must be defined for the state vector x and its error covariance matrix P at time t−1. However, their effect on the efficiency of the filter is not significant, as it has been proven that, very soon, both $x_{t}$ and $P_{t}$ converge to their actual values [32]. On the other hand, that is not the case with the covariance matrices $V_{t}$ and $W_{t}$, as the selected calculation method crucially affects the filter’s performance.

Researchers have developed several methods to update these quantities. Some studies apply covariance matrices that are fixed and defined prior to the usage of the filtering process [33,34], while others update them within the procedure using the past seven values of $w_{t}=x_{t}-x_{t-1}$ and $v_{t}=y_{t}-x_{t}$ [35,36]. Here, the initial strategy is applied.

### Non-Linear Kalman Filter

Through KF, this study aims to decode and thus eliminate the systematic error of the simulation, which is described as the difference between the observed measurement and the corresponding forecast from the wave numerical model WAM. Here, that bias ($y_{t}$) is expressed as a polynomial [19,37] of the model’s previous direct output ${SWH}_{t-1}$:

(10) $$y_{t}=x_{0,t}+x_{1,t}*SWH_{t-1}+x_{2,t}*{SWH_{t-1}}^{2}+\cdots +x_{n,t}*{SWH_{t-1}}^{m}+v_{t},$$

where m expresses the degree of the polynomial and n=m+1 is the dimension of the state vector.

This work proposes a quadric polynomial, i.e., m=2, as Bogdanovs et al. [37] observed that employing greater degrees of polynomials results in a substantial estimation error deviation. Therefore, Equation (10) is transformed to

$$y_{t}=x_{0,t}+x_{1,t}*SWH_{t-1}+x_{2,t}*{SWH_{t-1}}^{2}+v(t)$$

The equation above forms the measurement equation with state vector $x_{t}=[x_{0,t}x_{1,t}x_{2,t}]$ and measurement transition matrix $B_{t}=[1\;SWH_{t-1}\;{SWH_{t-1}}^{2}]$. Furthermore, regarding the progression of the state vector over time, it is assumed that its change is random due to the lack of accurate information; therefore, the system’s transition matrix is equal to $A_{t}=1$.

Based on the aforementioned, the system Equation (3) and the measurement Equation (4) for this study becomes

$$x_{t}={\left[x_{0,t-1}x_{1,t-1}x_{2,t-1}\right]}^{T}+w_{t}$$

and

$$y_{t}=[1SWH_{t-1}{SWH_{t-1}}^{2}\;]\;x_{t}+v_{t}$$

The initial value for the vector x at time t−1 is considered zero unless other indications about its prior condition are available, whereas its corresponding error covariance matrix P is set to be diagonal with relatively large values, which dictates low trust in the initial guesses. In particular, it is proposed that $P_{t-1}=\left[\begin{array}{ccc} 4 & 0 & 0\\ 0 & 4 & 0\\ 0 & 0 & 4 \end{array}\right]$ [25].

Crucial for the three-dimensional filter’s successful implementation is the selection of the covariance matrices. In general, a safe strategy is to assume initial values close to zero and later adaptively update and estimate them. However, as it is unclear which adaptation rule to apply, this study utilizes fixed covariance matrices that were defined before the use of the filter. Specifically, various tests are conducted with different combinations of $W_{t}$ and $V_{t}$ to determine the optimal one. The results show that for the environmental parameter of significant wave height, the best values were $V_{t}=4$ and $W_{t}=I_{3}$, respectively, where $I_{3}$ is the identity matrix.

When the filtering process is done, the systematic error of the simulation is obtained through the optimal state vector $x^{*}$, which is then added to WAM’s direct output to produce the “corrected” forecasts for the second stage of the dual filter (Radial Basis Function neural network implementation).

$${CorrectedSWH}_{t}={SWH}_{t}+B_{t}x^{*}$$

## 4. Radial Basis Function Neural Networks

While the polynomial variation of the non-linear Kalman filter algorithm is effective in mitigating systematic deviations, it struggles to address the stochastic and unpredictable nature of the remaining white noise. To overcome this obstacle, the proposed methodology sequentially combines the quadric KF with an RBF neural network, which acts as a secondary filter to constrain the non-systematic part of the forecast error.

**Figure 4:**
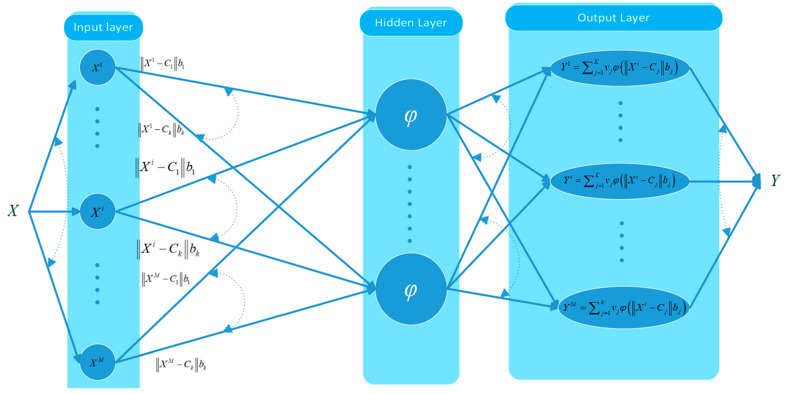
A standard Radial Basis Function neural network.

Radial Basis Function neural networks [38,39] are a special type of ANN that has been widely utilized in the academic community [40,41,42,43] due to their simple design and training algorithms, which are distinguished by their high accuracy and minimal computational cost [39]. A standard RBF structure consists of three layers: the input layer, the hidden layer with several neurons (clusters) and radial basis functions as activation functions (φ), and the linear output layer (Figure 4). 

Despite the simplicity of the architecture, choosing the activation function and the network’s parameters may be a difficult task. In terms of activation functions, this work employs the Gaussian [44,45,46], $\varphi \left(x\right)=e^{-x^{2}}$ and the Multiquadric [47,48,49], $\varphi \left(x\right)=\sqrt{1+x^{2}}$, as there are insufficient indications on which one is best suited to the wave parameter under study.

The major distinction between these transfer functions is their response. The Gaussian has a local response, which means that the neuron’s output is closer to zero if the distance from the center point increases, while the Multiquadric exhibits the opposite behavior and is therefore characterized by a global response. More information about their main properties can be found in Hagan et al. [17].

When the activation function is specified, the network’s parameters must be defined through the training process. Typically, there are two strategies for training an RBF neural network: the first approach applies non-linear, gradient-based optimization procedures to determine all the network parameters in one step [50,51], whereas the second approach divides the training process into two phases.

The first phase tries to determine the number and locations of the hidden node centroids, while the second phase specifies the synaptic weights. This two-stage procedure exploits the linear interconnection of the hidden and output layers, which allows the use of linear regression to calculate the weights [52]. Hence, it is frequently faster than optimizing all RBF network parameters simultaneously [53].

This study applies the two-stage approach. To demonstrate the training process, let’s present as $X^{i}={[X_{1}^{i},X_{2}^{i},\ldots ,\;X_{Q}^{i}]\;}^{T}$ the ith input vector of a Q×k matrix X, where k=1,…,i,…,M, with M being the number of training patterns and Q being the dimension of the input vectors.

Initially, the RBF network calculates the distance between the ith input vector and each centroid ($C_{j}$) in the hidden layer. Afterward, that outcome is multiplied by an offset parameter ($b_{j}$), known as width, which scales the activation function, instigating it to either widen or enlarge. As a result, the network input for the jth hidden layer neuron can be computed as

$${netinput}_{j}=||X^{i}-C_{j}||b_{j},$$

where ||∘|| represents the Euclidean distance.

The produced quantity is transformed via the transfer function (here, the Gaussian or the Multiquadric) and generates the output of the j neuron, which is then multiplied by the corresponding synaptic weight ($v_{j}$). Extending this process to each neuron in the hidden layer and summing up the results, the direct output of the RBF network is obtained by

(11) $$Y^{i}=\sum_{j=1}^{K}v_{j}\varphi (\left|\left|X^{i}-C_{j}\right|\right|b_{j}),$$

where K expresses the number of centroids.

The next step of the illustrated process is the determination of the locations of the hidden layer centers. Here, the Kmeans++ algorithm is implemented [54]. Kmeans++ is an improved version of the classic Kmeans [55] that identifies a set of centroids with an O(log⁡(q)) approximation for the optimum center set [56]. However, Kmeans++ does not instantly define the optimum number of clusters (neurons); instead, this quantity should be specified prior to applying the method, which creates uncertainty regarding their optimal value.

To avoid this major drawback and define the size of the network size, the proposed methodology trains the Radial Basis Function neural network for multiple clusters ranging from 10 to 70. Their optimal number would be the one that minimizes the Sum-Squared-Error (*SSE*):

(12) $$SSE=\sum_{k=1}^{M}{\left({er}^{k}\right)}^{2},$$

where ${er}^{k}$ is the kth training error, i.e., ${er}^{k}=(T^{k}-Y^{k})$, with $T^{k}$ being the corresponding scalar target for the kth input vector.

Based on the established centroids, the width of each cluster can be determined through the next formula [17]:

$$b_{j}=\frac{1}{\sqrt{2}{dist}_{j}}$$

where ${dist}_{j}$ presents the average distance between the associated center of the j cluster and its neighbors and is computed by

$${dist}_{j}=\frac{1}{{Inp}_{c}}\sqrt{\sum_{p=1}^{{Inp}_{c}}\left||X^{p}-C_{j}|\right|}.$$

Here, the quantity ${Inp}_{C}$ expresses the number of input vectors that are closest to the related center. Therefore, $X^{1}$ and $X^{2}$ are the nearest and the next nearest input vectors to the center $C_{j}$.

That concludes the first phase of the two-stage training algorithm. The next and final step includes the estimation, through linear regression, of the synaptic weights that connect the hidden with the output layer. To present this process, the network’s response for the matrix X based on Equation (11) is expressed as

Y=ΦV,

where $\Phi ={\left[\varphi ({Χ}^{k},C_{j},b_{j})\right]}_{i,j}\;(k=1,\ldots i,\ldots ,M,\;j=1,\ldots ,K)$ is the radial functions φ output matrix and $V={\left[v_{1},\ldots ,v_{K}\right]}^{T}$ is the synaptic weights vector. Thus, the vector of weights that optimizes the performance of the RBF architecture, i.e., minimizes Equation (12), is given by

$$\hat{V}={{(\Phi }^{Τ}\Phi )}^{-1}\Phi^{Τ}T,$$

where T presents the scalar target values of the M training patterns, i.e., $T={\left[T^{1},\ldots ,T^{i},\ldots ,T^{M}\right]}^{T}$.

Aside from the analysis of the training algorithm, another issue that needs to be clarified for the successful implementation of the RBF network is the treatment of overfitting. Overfitting is a phenomenon in which an ANN memorizes the properties of a known data set, inhibiting the formation of models that effectively extrapolate from observed to unseen data [57].

To address this issue, this work applies the L2 regularization strategy [52]. The primary aim of this procedure is to reduce the network’s dependency on specific “routes” not by decreasing the network weights but by constraining their magnitude. To accomplish this, a parameter λ>0 is added to the Sum-Squared-Error to penalize large weights. Hence, the SSE (12) is transformed into

(13) $$SSE=\sum_{k=1}^{M}{\left({er}^{k}\right)}^{2}+\lambda \sum_{j=1}^{K}v_{j}^{2}$$

and the corresponding optimal vector $\hat{V}$ is transformed to

$${\hat{V}}^{*}={{(\Phi }^{Τ}\Phi +\lambda {Ι}_{Κ})}^{-1}\Phi^{Τ}T$$

The determination of the penalty parameter λ is not an easy task, as its value will crucially affect the generalization capabilities of the RBF network; therefore, the choice cannot be random. Several approaches have been developed to define the λ parameter [58], but this work suggests an alternative strategy. More specifically, for every number of clusters, multiple trainings are conducted for different values of that parameter, ranging from $10^{-6}$ to $10^{-2}$. The ideal value is the one that minimizes Equation (13).

The developed dual filter is outlined in Algorithm 1, while the main characteristics of the Radial Basis Function neural network are summarized in Table 1. Detailed results about the number of clusters, penalty parameters, and activation functions from the RBF’s training process can be found in Appendix A.
**Algorithm 1: Combine KFs and RBFNNs**.Based on the training data set (Tr):{Inputs, Targets} → {Model’s Forecast, Observations}  **for** each element in Tr do
Apply the non-linear Kalman filter and obtain $x^{*}$

  **endfor**
Create the Input data for the RBF network${CorrectedSWH}_{t}={SWH}_{t}+B_{t}x^{*}$Create the training and validation datasets for the RBFDistinct training and validation datasets for each training. Same for every topology**for** each Cluster do   **for** each penalty parameter do    **for** each Activation function do       Form the RBF structure.% number of clusters, regularization parameter λ, activation function      **while** train ≤ maxtrain % Conduct multiple trainings for each structure       Determine the centroids from the training dataset using the K-means++ and compute the widths.       Train network using LLS based on the training data set.        performance → Network’s performance % SSE based on the validation data set          **if** performance < Initial Value           Set Initial Value equal to performance           The best results for every combination are stored in a cell array. Number of clusters, performance, penalty parameter, activation function, train time, best centers, widths, and external weights.          **endif**       train → train+1      **endwhile**      train → 1    **endfor**   **endfor**   readjust Initial Value**Endfor****Define the optimal RBF network structure**  **if** several indices in the Total SSE vector display similar results. % Their absolute difference remains smaller than a specified threshold  position → the index with minimum train time  **else**  position → the minimum SSE index  **end**Best RBFNN structure → best results{position}. 

The produced dual filter is constructed primarily as a self-adaptive computational system that simultaneously targets the systematic and non-systematic parts of the forecast error. Nevertheless, the proposed method indirectly can also boost the computational efficiency of WAM, by minimizing the requirement for high-resolution simulations, or repeated runs of the numerical wave prediction model. That is partly owing to the use of Kalman filters and Radial Basis Function neural networks, which are highly efficient post-processing techniques. Their relatively low computational cost, along with their capacity to generate enhanced predictions, makes the overall framework efficient compared to original numerical models. Therefore, the developed dual filter accomplishes significant error reductions without increasing the computational demands of the core model.

## 5. Case Study

This section illustrates the time window process implementation for the various regions and time periods in the Aegean Sea (Mykonos and Crete) and the Pacific Ocean (46002). Particularly, the suggested dual filter was utilized for forecasting significant wave height, concerning the areas of Mykonos and Heraklion from 2007 to 2009 and the region 46002 between 2011 and 2013. The purpose of this procedure is double. First, the suggested approach’s stability is tested over different locations and time periods, and later, the combined filter is compared to the classic Kalman filter through a range of assessment indicators and time series diagrams.

Algorithm 1 is applied in each time window using predefined training data sets (Training data set) and testing intervals (Testing data set), which are determined before the application of the process. These hyperparameters are not chosen randomly but rather after a series of sensitivity experiments, the results of which are presented in Table 2.

Table 2 clearly shows that no fixed Training data set exists for the dual filter process that describes all the locations under study, and the same is true for the Testing data set. However, this is not the situation concerning the range of Time Windows, since the conducted tests revealed that the ideal value is five for every case study. It is important to highlight at this point that the new recorded observations are available via the stations presented in Section 2 every three hours.

The suggested Time-Window Process is outlined in Algorithm 2.
**Algorithm 2: Time-Window Process**Data loading:{Inputs, Targets} → {Model’s Forecast, Observations}Data normalization: {Inputs, Targets} → [−1,1]Determination of the Training and Testing data setsConcerns the training process of the dual filter (KF and RBF) and the evaluation of the methodDetermine the time window’s maximum number:Max_tw=Time Window−1Set the appropriate matrices and vectors for storing the outcomes 
Determine the set of penalty parameters:Penalty Parameter λ$\;\to \;[10^{-6},10^{-2}]$Determine the set of Clusters for the RBF network:Cluster → [10:step:70]Determine the set of activation functions:Activation function → {Gaussian, Multiquadric} 
**for** qq=0,…,Max_tw

  Tr →(1+qq):Training Data+qq
Training data for the RBFNN.% One step in time.
  Run the Algorithm 1 Obtain the optimal RBF structure. Centroids, widths, external weights, activation fun. 
${\;\;Tr}_{1}$ → $Tr\left(end\right)+1:Tr\left(end\right)+TestingData$
Testing data for the improved forecasts and the evaluation.
  Denormalize the data Improved forecasts, corresponding model forecasts, and recorded observations.
  Assess the method based on ${Tr}_{1}$ and store the resultsBias, Absolute Bias, Rmse, and Ns indices.
**End**Save results from each time window


### 5.1. Method’s Evaluation

To analyze the efficiency of the integrated method, the following assessment indices were used:

- Bias of the forecasting values:

$$Bias=\frac{\sum_{t=1}^{N}obs(t)-for(t)}{N}$$

Bias is an important aspect of filtering processes since it offers information about systematic inaccuracy, namely whether the model overestimates or underestimates the actual observations. Ideal value: Bias=0.

- Absolute Bias of the forecasting values:

$$AbsoluteBias=\frac{\sum_{t=1}^{N}\left|obs(t)-for(t)\right|}{N}\;$$

The Absolute Bias does not negate positive and negative deviations and cannot identify the type of error (overestimated or underestimated), thus it should be used in conjunction with Bias. Ideal value: Absolute Bias=0.

- Root Mean Square Error:

$$Rmse=\sqrt{\frac{\sum_{t=1}^{N}{\left(obs(t)-for(t)\right)}^{2}}{N}}$$

Rmse is a crucial factor for any filtering procedure, as it measures the variability of the error and reflects the overall predictive performance. Ideal value: Rmse=0.

- Nash-Sutcliffe efficiency coefficient:

(14) $$Ns=1-\frac{\sum_{t=1}^{N}{\left(for(t)-obs(t)\right)}^{2}}{\sum_{t=1}^{N}{\left(obs(t)-\bar{obs}\right)}^{2}}$$

The Nash–Sutcliffe efficiency coefficient for the model fluctuates in (−∞,1). The value 1 indicates a flawless model, as its predictions match the observations perfectly while obtaining value zero implies that the accuracy of the model is as good as the accuracy of the reference model (here the mean value of observations, $\bar{obs}$). If the value of the Ns is negative, the accuracy of the model is worse than the accuracy of the reference model. Ideal value: Ns=1.

For every indicator, the parameter N expresses the size of the Testing data set (model’s forecasts & observations), obs(t) presents the observed value and for(t) the corresponding model’s forecast, or the enhanced prediction of the suggested methodology, at time t.

### 5.2. Results

**Figure 5:**
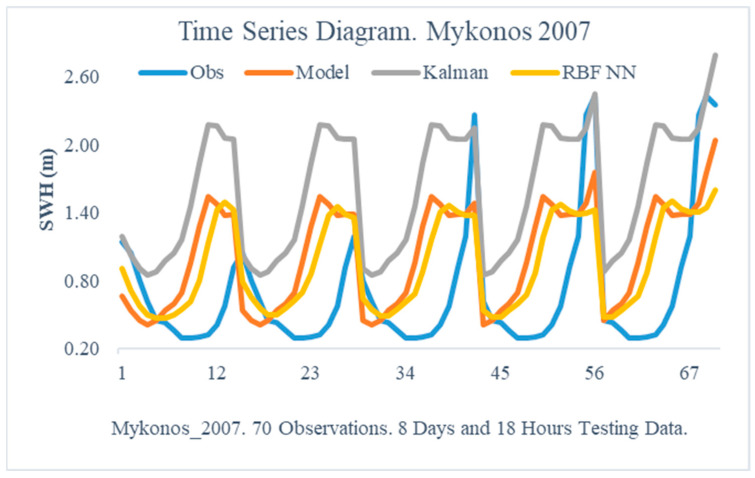
Time Series Diagram. Mykonos 2007.

**Figure 6:**
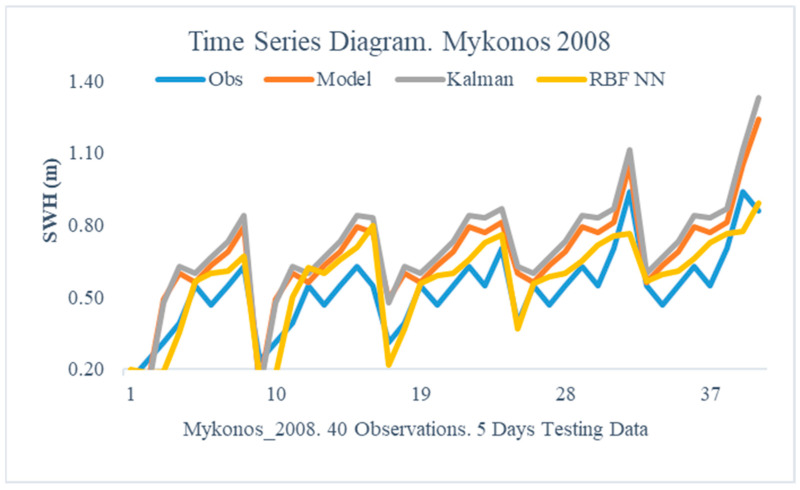
Time Series Diagram. Mykonos 2008.

**Figure 7:**
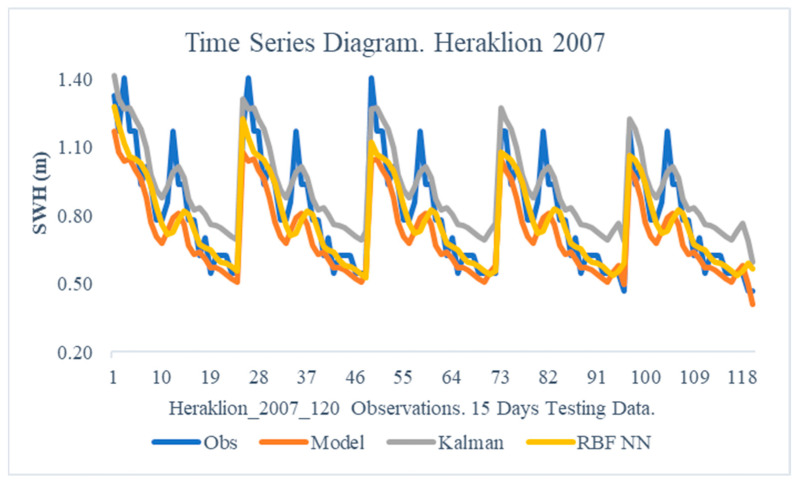
Time Series Diagram. Heraklion 2007.

**Figure 8:**
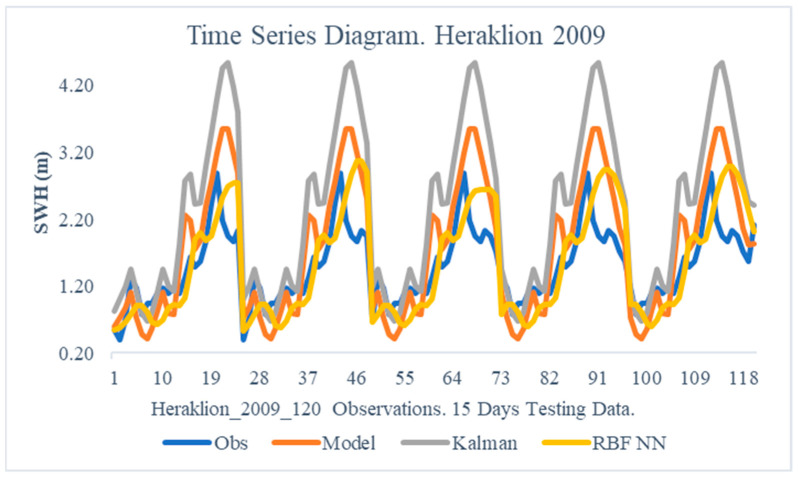
Time Series Diagram. Heraklion 2009.

**Figure 9:**
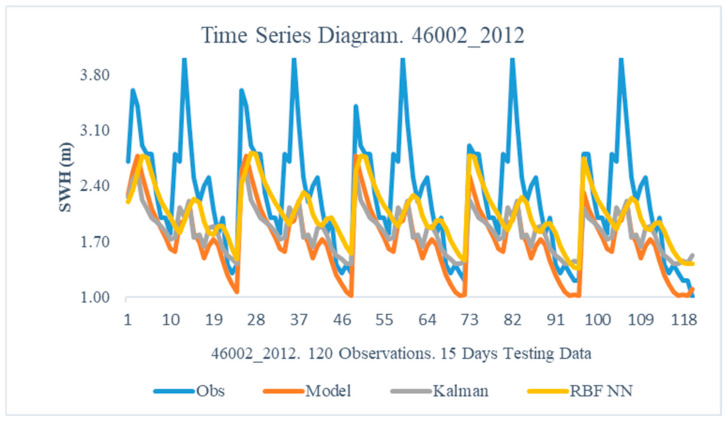
Time Series Diagram. 46002 2012.

**Figure 10:**
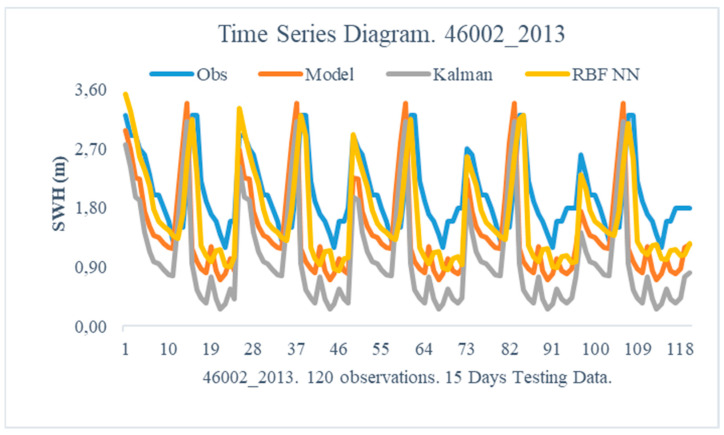
Time Series Diagram. 46002 2013.

The obtained results from the proposed method are illustrated in this section. In particular, the following Figure 5, Figure 6, Figure 7, Figure 8, Figure 9 and Figure 10 demonstrate the aggregate time series diagrams for each time window, with x-axis presenting the total forecasts, while in the Table 3, Table 4 and Table 5 below, the corresponding (average) values of the statistical indices are recorded. Extensive analysis for every time window can be found in Appendix B and Appendix C, respectively.

#### 5.2.1. Time-Window Process: Region of Mykonos, Aegean Sea

The analysis of the findings from the Mykonos region reveals that the suggested approach significantly improves the predictions of the wave numerical model WAM. In 2007, every evaluation indicator (Table 3) was considerably improved, with the minimum increase being 15% for the Rmse and the largest improvement exceeding 50%. In contrast, examining the comparable values from the Kalman filter implementation reveals that the traditional methodology fails to enhance the simulation system’s predictions. The Rmse indicator grew significantly (1.0747 from 0.6521—Table 3 Mykonos 2007), signifying that the KF was unable to detect and, thus, reduce the associated forecast uncertainty.

Greater was the contribution of the dual filter in 2008. During this period, the Bias and Absolute Bias dropped by 72% and 43%, respectively, while the Rmse index decreased by more than 35%. On the contrary, KF’s usage provides no improvements as each rating assessor deteriorated significantly, culminating in the Ns index, which decreased from −0.3792 to −1.075 (Table 3—Mykonos 2008).

The time series graphs (Figure 5 and Figure 6) show the same results. In particular, Figure 6 illustrates that the predictions derived using the suggested method have a nearly identical distribution to the actual observations. Hence, in the area of Mykonos, the combined approach not only improves WAM forecasts but also avoids the constraints of the standard Kalman filter.

#### 5.2.2. Time-Window Process: Region of Heraklion, Aegean Sea

The obtained results from the area of Heraklion show that the dual filter based on the RBF network structure improves the forecasting capabilities of the NWP system considerably. Specifically, in 2007 (Table 4), the combined approach managed to decode the systematic part of the forecast error as the Bias index decreased by 60%. Furthermore, the Rmse indicator for the same period was reduced by 26%, which indicates that the suggested methodology was able to detect the variability in the remaining non-systematic part of the forecast error. On the other hand, the corresponding results derived from the classic Kalman filter implementation worsened the predictive ability of the numerical model, as the Bias and Absolute Bias increased by 3% and 16%, respectively.

To a greater extent, the improvement was caused by the application of the proposed method for the year 2009 (Table 4). Particularly, the Bias and Absolute Bias indices decreased by 83% and 20%, respectively, while the Rmse indicator decreased by 23%. Yet again, the standard Kalman filter was unable to enhance the forecasts of the wave model in use as two crucial assessors, the Bias and Rmse, increased by almost 66% and 78%, respectively.

Comparable conclusions can be drawn by analyzing the time series diagrams (Figure 7 and Figure 8), as the dual filter provides stable final forecasts that lead to an accurate convergence of the modeled PDFs to the observations. Therefore, for the Heraklion region, the combined use of Kalam filters and Radial Basis Function neural networks improves the WAM’s forecasts.

#### 5.2.3. Time-Window Process: Region 46002, Pacific Ocean

The dual filter’s consistent behavior remains in the Pacific Ocean case. According to the derived results (Table 5—46002 2012), both the proposed methodology and the standard Kalman filter improve the predicting abilities of the simulation model being used. However, the combination of Kalman filters and Radial Basis neural networks yields better results, as the Bias Index decreased by 59%, whereas the corresponding reduction from the KF was 19%.

Moving on to the next period, only the proposed methodology successfully improves the WAM model’s predictions. Specifically, the Bias and Absolute Bias indices were reduced from 0.5797 to 0.3215 and from 0.8272 to 0.3945, respectively (Table 5—46002 2013). The Rmse indicator showed a considerable improvement of more than 50%, implying that the proposed approach limits the variability in the remaining non-systematic part of the forecast error, resulting in more accurate final forecasts.

These conclusions can also be obtained from the time series diagrams (Figure 9 and Figure 10). Studying Figure 9, it seems that both the combined approach and the standard Kalman filter appear to better capture the morphology of the recorded observation than the initial model. Figure 10 demonstrates the superiority of the dual filter, as the produced forecasts are closer to the recorded observations during a fifteen-day forecast interval. As a result, combining Kalam filters and Radial Basis Function neural networks improves the WAM model’s predictive capacity.

To summarize the results obtained from the time-window process, the dual filter successfully improves the WAM model’s forecasts in all cases, regardless of the period or geographic location. However, it is important to mention that when the focus is on the Aegean Sea, the produced system tends to overestimate the recorded observations. In contrast, in the Pacific Ocean regions, the combined post-processing algorithm tends to underestimate them. That diverge behavior is due to the complexity of the significant wave height and the WAM’s physical parametrization.

On the one hand, SWH’s prediction is challenging due to its dependence on non-linear processes such as wave-wave interactions, energy transfer, and dissipation mechanisms like white capping, which are difficult to represent accurately in models. On the other hand, WAM relies on parameterized representations of wave dynamics, which, while computationally efficient, can oversimplify the complexities of real-world phenomena, leading to deviations between predicted and observed wave conditions, particularly in complex or extreme environments. One example is the underestimation of the observed peak values in Figure 9 and the underestimation of the observed minimum values in Figure 10. Still, though, the dual filter manages to overcome these limitations and produce superior predictions compared to the ones produced by WAM.

Finally, it is revealed that the standard KF cannot enhance the final predictions of the wave numerical model. This lack of improvement observed with the classical Kalman filtering method is partly due to its restricted capabilities in addressing the non-systematic error. This part of the forecast error, which increases the variability and unpredictability of final predictions, cannot be effectively addressed through the bias adjustment process of the KF algorithm. As a result, increased RMSE and bias values are recorded in the Kalman filter results, especially during complex or highly dynamic environmental conditions.

## 6. Conclusions

The motivation of this research was to develop a novel post-processing algorithm that combines Radial Basis Function neural networks and Kalman filters to improve the forecasts of a numerical wave model regarding the parameter of significant wave height. To accomplish this, the produced model targets the simulation’s systematic error alongside the remaining non-systematic part of that error.

Initially, a non-linear Kalman filter is applied to decode and, as a result, eliminate the bias between the recorded observations and the direct outputs of the WAM system. Afterward, a Radial Basis Function neural network is utilized, acting as an additional filter, with the goal of detecting and reducing the variability in the non-systematic part of that bias and the accompanying anticipated uncertainty.

The suggested methodology was applied via a time-window process involving several regions and time periods. The first case study concerns the areas of Mykonos and Heraklion (Crete) in the Aegean Sea from 2007 to 2009, while the second case focuses on the region 46002 in the Pacific Ocean between 2011 and 2013. For every case study, the extracted results were compared to those obtained by the classic Kalman filter to determine the degree of improvement offered by the suggested dual filter.

The results revealed that combining RBF neural networks and KFs significantly improved the forecasting capabilities of the simulation system in use. Specifically, the recorded systematic errors decreased considerably, with an average reduction of 53% in the Bias index, whereas the Rmse evaluation indicator and, thus, the related forecast uncertainty were reduced by 28%. In contrast, the standard Kalman filter implementation resulted in a 73% and 37% increase in the relevant indices.

Furthermore, the usage of Kalman filters in conjunction with Radial Basis Function neural networks illustrated stable behavior regardless of forecasting horizons and geographical regions, providing a smooth and efficient tool that avoids the boundaries of classic Kalman filters, which substitute initial systematic deviations with comparable over- and under-estimation periods, leading to lower mean error values but no meaningful gain in forecasts.

The suggested methodology is applicable to similar simulations in fields such as economics or signal processing, as it is independent of the type of data and therefore can be extended beyond environmental applications.

## Figures and Tables

**Table 1:** Properties of the Radial Basis Function neural network.

ANN	Radial Basis Function Neural Network
Clusters	10 to 70 with step=10
Clustering Method	Kmeans++
Activation Function	Gaussian or Multiquadric
Hidden Layers	One
Output Layer	One Linear Output
Training Algorithm	Two-stage
Overfitting	Regularization
Penalty Parameter (λ)	$\lambda \;\in [10^{-6},10^{-2}]$

**Table 2:** Hyperparameters of the Time-Window Process.

**SWH Aegean Sea**	**Time Windows**	**Training Data**	**Testing Data**
Mykonos 2007	5	350	14
Mykonos 2008	5	510	8
Heraklion 2007	5	360	24
Heraklion 2009	5	300	24
**SWH Pacific Ocean**	**Time Windows**	**Training Data**	**Testing Data**
46002 2012	5	450	24
46002 2013	5	250	24

**Table 3:** Time-Window Process. Average results from the area of Mykonos.

**Average Results Mykonos 2007**	**Bias**	**AbsoluteBias**	**Rmse**	**Ns**
Model	−0.3298	0.5408	0.6521	−1.6190
Kalman	−0.8882	0.8995	1.0747	−6.2782
RBF	−0.2472	0.4482	0.5575	−0.6924
**Average Results Mykonos 2008**	**Bias**	**AbsoluteBias**	**Rmse**	**Ns**
Model	−0.1336	0.1463	0.1624	−0.3792
Kalman	−0.1699	0.1850	0.1996	−1.0756
RBF	−0.0380	0.0834	0.0996	0.4518

**Table 4:** Time-Window Process. Average results from the area of Heraklion.

**Average Results_Heraklion 2007**	**Bias**	**AbsoluteBias**	**Rmse**	**Ns**
Model	0.0996	0.1075	0.1389	0.6346
Kalman	−0.1028	0.1247	0.1379	0.6285
RBF	0.0398	0.0794	0.1232	0.7110
**Average Results_Heraklion 2009**	**Bias**	**AbsoluteBias**	**Rmse**	**Ns**
Model	−0.2863	0.5270	0.6563	−0.4418
Kalman	−0.8454	0.8846	1.1686	−3.5855
RBF	−0.0476	0.4204	0.5039	0.1252

**Table 5:** Time-Window Process. Average results from the area of 46002.

**Average Results_46002 2012**	**Bias**	**AbsoluteBias**	**Rmse**	**Ns**
Model	0.5546	0.5563	0.7081	−0.0184
Kalman	0.4516	0.5142	0.6800	0.0594
RBF	0.2288	0.4192	0.6100	0.2408
**Average Results_46002 2013**	**Bias**	**AbsoluteBias**	**Rmse**	**Ns**
Model	0.5797	0.8272	0.9619	−1.9924
Kalman	0.9753	1.1353	1.2478	−4.0453
RBF	0.3215	0.3945	0.4810	0.2488

**Figure A1:**
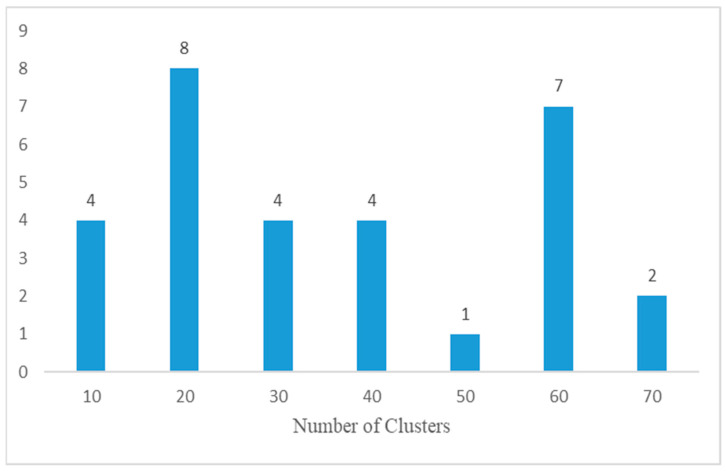
Number of clusters. Total results for 30 time windows.

**Figure A2:**
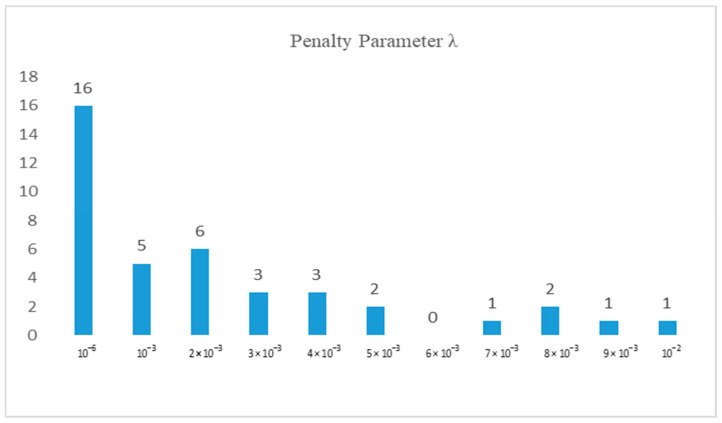
Regularization parameter. Total results for 30 time windows.

**Figure A3:**
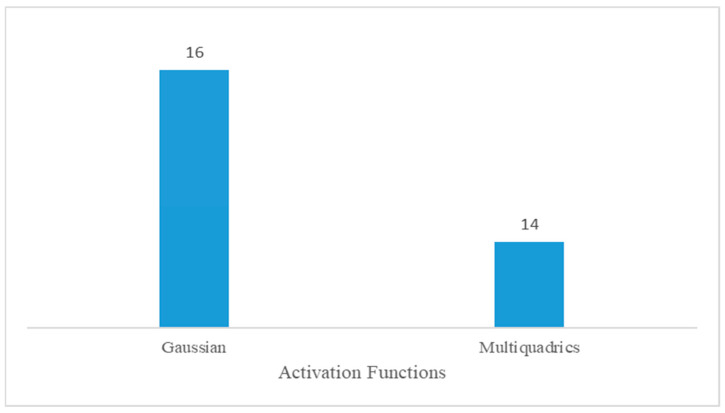
Activation functions. Total results for the 30 time windows.

## Data Availability

For privacy reasons, the data presented in this study can be obtained upon request from the corresponding author.

## Appendix B. Results for the Aegean Sea

**Table A1:** Results from the area of Mykonos 2007.

	**Time Window 1**						**Time Window 4**				
	**Bias**	**AbsoluteBias**	**Rmse**	**Ns**	**TrainTime**		**Bias**	**AbsoluteBias**	**Rmse**	**Ns**	**TrainTime**
Model	−0.3136	0.5342	0.6409	−4.1342		Model	−0.3194	0.5569	0.6681	0.0603	
Kalman	−0.8454	0.8479	1.0537	−12.8753		Kalman	−0.8900	0.9072	1.0787	−1.4499	
RBF	−0.2579	0.3869	0.4966	−2.0825	0.0002	RBF	−0.2142	0.4944	0.6109	0.2144	0.0002
	**Time Window 2**						**Time Window 5**				
	**Bias**	**AbsoluteBias**	**Rmse**	**Ns**	**TrainTime**		**Bias**	**AbsoluteBias**	**Rmse**	**Ns**	**TrainTime**
Model	−0.3624	0.5139	0.6301	−3.7199		Model	−0.3116	0.5647	0.6711	0.2915	
Kalman	−0.9039	0.9064	1.0786	−12.8332		Kalman	−0.9051	0.9223	1.0834	−0.8466	
RBF	−0.3133	0.3804	0.4858	−1.8054	0.0001	RBF	−0.1755	0.5455	0.6456	0.3442	0.0003
	**Time Window 3**										
	**Bias**	**AbsoluteBias**	**Rmse**	**Ns**	**TrainTime**						
Model	−0.3423	0.5340	0.6503	−0.5928							
Kalman	−0.8965	0.9137	1.0791	−3.3859							
RBF	−0.2753	0.4340	0.5484	−0.1326	0.0004						

**Table A2:** Results from the area of Mykonos 2008.

	**Time Window 1**						**Time Window 4**				
	**Bias**	**AbsoluteBias**	**Rmse**	**Ns**	**TrainTime**		**Bias**	**AbsoluteBias**	**Rmse**	**Ns**	**TrainTime**
Model	−0.0899	0.1274	0.1423	0.1590		Model	−0.1416	0.1416	0.1546	0.0177	
Kalman	−0.1078	0.1532	0.1662	−0.1461		Kalman	−0.1877	0.1877	0.1978	−0.6089	
RBF	−0.0103	0.0632	0.0737	0.7746	0.0003	RBF	−0.0285	0.0779	0.1004	0.5858	0.0002
	**Time Window 2**						**Time Window 5**				
	**Bias**	**AbsoluteBias**	**Rmse**	**Ns**	**TrainTime**		**Bias**	**AbsoluteBias**	**Rmse**	**Ns**	**TrainTime**
Model	−0.1235	0.1495	0.1619	−0.6413		Model	−0.1631	0.1631	0.1913	−0.5047	
Kalman	−0.1508	0.1809	0.1927	−1.3255		Kalman	−0.2170	0.2170	0.2446	−1.4610	
RBF	−0.0675	0.1202	0.1318	−0.0879	0.0004	RBF	−0.0432	0.0849	0.1038	0.5567	0.0003
	**Time Window 3**										
	**Bias**	**AbsoluteBias**	**Rmse**	**Ns**	**TrainTime**						
Model	−0.1499	0.1499	0.1621	−0.9266							
Kalman	−0.1864	0.1864	0.1967	−1.8365							
RBF	−0.0407	0.0709	0.0882	0.4299	0.0003						

**Table A3:** Results from the area of Heraklion 2007.

	**Time Window 1**						**Time Window 4**				
	**Bias**	**AbsoluteBias**	**Rmse**	**Ns**	**TrainTime**		**Bias**	**AbsoluteBias**	**Rmse**	**Ns**	**TrainTime**
Model	0.1152	0.1203	0.1508	0.6393		Model	0.0867	0.0974	0.1259	0.6518	
Kalman	−0.0913	0.1178	0.1299	0.7324		Kalman	−0.1137	0.1287	0.1430	0.5507	
RBF	0.0523	0.0853	0.1297	0.7334	0.0002	RBF	0.0274	0.0732	0.1150	0.7096	0.0002
	**Time Window 2**						**Time Window 5**				
	**Bias**	**AbsoluteBias**	**Rmse**	**Ns**	**TrainTime**		**Bias**	**AbsoluteBias**	**Rmse**	**Ns**	**TrainTime**
Model	0.1085	0.1138	0.1473	0.6256		Model	0.0841	0.0948	0.1240	0.6355	
Kalman	−0.0956	0.1220	0.1343	0.6890		Kalman	−0.1146	0.1296	0.1437	0.5104	
RBF	0.0500	0.0839	0.1279	0.7177	0.0001	RBF	0.0214	0.0738	0.1158	0.6821	0.0002
	**Time Window 3**										
	**Bias**	**AbsoluteBias**	**Rmse**	**Ns**	**TrainTime**						
Model	0.1033	0.1114	0.1463	0.6210							
Kalman	−0.0988	0.1252	0.1385	0.6600							
RBF	0.0479	0.0810	0.1275	0.7120	0.0003						

**Table A4:** Results from the area of Heraklion 2009.

	**Time Window 1**						**Time Window 4**				
	**Bias**	**AbsoluteBias**	**Rmse**	**Ns**	**TrainTime**		**Bias**	**AbsoluteBias**	**Rmse**	**Ns**	**TrainTime**
Model	−0.2678	0.5076	0.6476	−0.1672		Model	−0.2935	0.5333	0.6589	−0.6631	
Kalman	−0.7882	0.8275	1.1287	−2.5454		Kalman	−0.8712	0.9105	1.1867	−4.3944	
RBF	0.0252	0.3469	0.4184	0.5129		RBF	−0.0925	0.4750	0.5589	−0.1963	0.0001
	**Time Window 2**						**Time Window 5**				
	**Bias**	**AbsoluteBias**	**Rmse**	**Ns**	**TrainTime**		**Bias**	**AbsoluteBias**	**Rmse**	**Ns**	**TrainTime**
Model	−0.2892	0.5290	0.6575	−0.2790		Model	−0.2914	0.5354	0.6598	−0.6072	
Kalman	−0.8346	0.8739	1.1625	−2.9989		Kalman	−0.8785	0.9178	1.1881	−4.2117	
RBF	−0.0377	0.4269	0.5309	0.1660	0.0002	RBF	−0.1169	0.4590	0.5553	−0.1386	0.0001
	**Time Window 3**										
	**Bias**	**AbsoluteBias**	**Rmse**	**Ns**	**TrainTime**						
Model	−0.2897	0.5295	0.6577	−0.4924							
Kalman	−0.8542	0.8935	1.1768	−3.7773							
RBF	−0.0161	0.3944	0.4562	0.2820	0.0002						

## Appendix C. Results for the Pacific Ocean

**Table A5:** Results from the area of 46002 2012.

	**Time Window 1**						**Time Window 4**				
	**Bias**	**AbsoluteBias**	**Rmse**	**Ns**	**TrainTime**		**Bias**	**AbsoluteBias**	**Rmse**	**Ns**	**TrainTime**
Model	0.5913	0.5913	0.7327	−0.1631		Model	0.5325	0.5325	0.6903	0.0174	
Kalman	0.5322	0.5583	0.7153	−0.1084		Kalman	0.4074	0.4827	0.6532	0.1201	
RBF	0.3474	0.4674	0.6692	0.0297	0.0002	RBF	0.2009	0.3761	0.5797	0.3071	0.0002
	**Time Window 2**						**Time Window 5**				
	**Bias**	**AbsoluteBias**	**Rmse**	**Ns**	**TrainTime**		**Bias**	**AbsoluteBias**	**Rmse**	**Ns**	**TrainTime**
Model	0.5858	0.5858	0.7302	−0.0459		Model	0.5129	0.5213	0.6864	0.0885	
Kalman	0.5089	0.5450	0.7101	0.0108		Kalman	0.3575	0.4764	0.6474	0.1893	
RBF	0.2314	0.4440	0.6263	0.2304	0.0006	RBF	0.1621	0.3976	0.5836	0.3411	0.0002
	**Time Window 3**										
	**Bias**	**AbsoluteBias**	**Rmse**	**Ns**	**TrainTime**						
Model	0.5504	0.5504	0.7007	0.0109							
Kalman	0.4518	0.5085	0.6739	0.0851							
RBF	0.2020	0.4106	0.5914	0.2955	0.0002						

**Table A6:** Results from the area of 46002 2013.

	**Time Window 1**						**Time Window 4**				
	**Bias**	**AbsoluteBias**	**Rmse**	**Ns**	**TrainTime**		**Bias**	**AbsoluteBias**	**Rmse**	**Ns**	**TrainTime**
Model	0.5363	0.7838	0.9349	−1.1975		Model	0.5958	0.8433	0.9718	−2.3847	
Kalman	0.9154	1.0755	1.2023	−2.6341		Kalman	1.0000	1.1600	1.2667	−4.7504	
RBF	0.2557	0.3631	0.4520	0.4864	0.0003	RBF	0.3451	0.4082	0.4981	0.1109	0.0001
	**Time Window 2**						**Time Window 5**				
	**Bias**	**AbsoluteBias**	**Rmse**	**Ns**	**TrainTime**		**Bias**	**AbsoluteBias**	**Rmse**	**Ns**	**TrainTime**
Model	0.5683	0.8158	0.9558	−1.6385		Model	0.6000	0.8475	0.9740	−2.7323	
Kalman	0.9575	1.1175	1.2350	−3.4052		Kalman	1.0081	1.1682	1.2723	−5.3689	
RBF	0.2779	0.3771	0.4657	0.3736	0.0003	RBF	0.3552	0.4053	0.4712	0.1263	0.0002
	**Time Window 3**										
	**Bias**	**AbsoluteBias**	**Rmse**	**Ns**	**TrainTime**						
Model	0.5979	0.8454	0.9731	−2.0089							
Kalman	0.9952	1.1553	1.2629	−4.0680							
RBF	0.3738	0.4188	0.5182	0.1467	0.0001						

## References

[1] Takahashi K., Miyoshi Y., Balasis G., Daglis I.A., Mann I.R. (2016). Introduction to Wave-Particle Interactions and Their Impact on Energetic Particles in Geospace. Waves, Particles, and Storms in Geospace.

[2] Galanis G., Emmanouil G., Chu P.C., Kallos G. (2009). A New Methodology for the Extension of the Impact of Data Assimilation on Ocean Wave Prediction. Ocean. Dyn..

[3] Famelis I., Galanis G., Ehrhardt M., Triantafyllou D. (2014). Classical and Quasi-Newton Methods for a Meteorological Parameters Prediction Boundary Value Problem. Appl. Math. Inf. Sci..

[4] Famelis I.T., Tsitouras C. (2015). Quadratic shooting solution for an environmental parameter prediction problem. FJAM.

[5] Dong R., Leng H., Zhao C., Song J., Zhao J., Cao X. (2023). A Hybrid Data Assimilation System Based on Machine Learning. Front. Earth Sci..

[6] Rojas-Campos A., Wittenbrink M., Nieters P., Schaffernicht E.J., Keller J.D., Pipa G. (2023). Postprocessing of NWP Precipitation Forecasts Using Deep Learning. Weather Forecast..

[7] Krasnopolsky V. (2023). Review: Using Machine Learning for Data Assimilation, Model Physics, and Post-Processing Model Outputs. https://repository.library.noaa.gov/view/noaa/50158.

[8] Kordatos I., Donas A., Galanis G., Famelis I., Alexandridis A. (2024). Significant Wave Height Prediction in Nested Domains Using Radial Basis Function Neural Networks. Ocean. Eng..

[9] Kariniotakis G.N., Pinson P. Evaluation of the MORE-CARE Wind Power Prediction Platform. Performance of the Fuzzy Logic Based Models. Proceedings of the EWEC 2003—European Wind Energy Conference.

[10] Kariniotakis G., Martí I., Casas D., Pinson P., Nielsen T.S., Madsen H., Giebel G., Usaola J., Sanchez I. What Performance Can Be Expected by Short-Term Wind Power Prediction Models Depending on Site Characteristics?. Proceedings of the EWC 2004 Conference.

[11] Vanem E. (2011). Long-Term Time-Dependent Stochastic Modelling of Extreme Waves. Stoch. Environ. Res. Risk Assess..

[12] Giebel G. (2001). On the Benefits of Distributed Generation of Wind Energy in Europe. https://www.osti.gov/etdeweb/biblio/20246798.

[13] Resconi G. (2009). Geometry of Risk Analysis (Morphogenetic System). Stoch. Environ. Res. Risk Assess..

[14] Setoodeh P., Habibi S., Haykin S. (2022). Nonlinear Filters: Theory and Applications.

[15] Kalnay E. (2002). Atmospheric Modeling, Data Assimilation and Predictability.

[16] Pelland S., Galanis G., Kallos G. (2013). Solar and Photovoltaic Forecasting through Post-processing of the Global Environmental Multiscale Numerical Weather Prediction Model. Prog. Photovolt..

[17] Hagan M.T., Demuth H.B., Beale M.H., De Jésus O. (2014). Neural Network Design.

[18] Louka P., Galanis G., Siebert N., Kariniotakis G., Katsafados P., Pytharoulis I., Kallos G. (2008). Improvements in Wind Speed Forecasts for Wind Power Prediction Purposes Using Kalman Filtering. J. Wind. Eng. Ind. Aerodyn..

[19] Pelosi A., Medina H., Van Den Bergh J., Vannitsem S., Chirico G.B. (2017). Adaptive Kalman Filtering for Postprocessing Ensemble Numerical Weather Predictions. Mon. Wea. Rev..

[20] Delle Monache L., Nipen T., Liu Y., Roux G., Stull R. (2011). Kalman Filter and Analog Schemes to Postprocess Numerical Weather Predictions. Mon. Weather. Rev..

[21] Group T.W. (1988). The WAM Model—A Third Generation Ocean Wave Prediction Model. J. Phys. Oceanogr..

[22] Watson K.M., West B.J. (1975). A Transport-Equation Description of Nonlinear Ocean Surface Wave Interactions. J. Fluid Mech..

[23] Ardhuin F., Rogers E., Babanin A.V., Filipot J.-F., Magne R., Roland A., Van Der Westhuysen A., Queffeulou P., Lefevre J.-M., Aouf L. (2010). Semiempirical Dissipation Source Functions for Ocean Waves. Part I: Definition, Calibration, and Validation. J. Phys. Oceanogr..

[24] Bidlot J.-R. (2012). Present Status of Wave Forecasting at ECMWF.

[25] Emmanouil G., Galanis G., Kallos G. (2012). Combination of Statistical Kalman Filters and Data Assimilation for Improving Ocean Waves Analysis and Forecasting. Ocean. Model..

[26] Zodiatis G., Lardner R., Nikolaidis M., Sofianos S., Vervantis V., Zhuk E., Spanoudaki K., Kampanis N., Kallos G., Sylaios G. The new CYCOFOS forecasting at coastal, sub-regional and regional scales in the Mediterranean and the Black Sea. Proceedings of the EGU General Assembly 2021.

[27] Zodiatis G., Galanis G., Kallos G., Nikolaidis A., Kalogeri C., Liakatas A., Stylianou S. (2015). The Impact of Sea Surface Currents in Wave Power Potential Modeling. Ocean. Dyn..

[28] Janssen P.A.E.M., Onorato M. (2007). The Intermediate Water Depth Limit of the Zakharov Equation and Consequences for Wave Prediction. J. Phys. Oceanogr..

[29] Bidlot J.-R., Janssen P., Abdalla S. (2007). A Revised Formulation of Ocean Wave Dissipation and Its Model Impact.

[30] Welch G., Bishop G. An Introduction to the Kalman Filter. 2006. https://www.cs.unc.edu/~welch/media/pdf/kalman_intro.pdf.

[31] Revach G., Shlezinger N., Van Sloun R.J.G., Eldar Y.C. Kalmannet: Data-Driven Kalman Filtering. Proceedings of the ICASSP 2021—2021 IEEE International Conference on Acoustics, Speech and Signal Processing (ICASSP).

[32] Wang B., Sun Z., Jiang X., Zeng J., Liu R. (2023). Kalman Filter and Its Application in Data Assimilation. Atmosphere.

[33] Homleid M. (1995). Diurnal Corrections of Short-Term Surface Temperature Forecasts Using the Kalman Filter. Weather Forecast..

[34] Libonati R., Trigo I., DaCamara C.C. (2008). Correction of 2 M-Temperature Forecasts Using Kalman Filtering Technique. Atmos. Res..

[35] Xu J., Xiao Z., Lin Z., Li M. (2021). System Bias Correction of Short-Term Hub-Height Wind Forecasts Using the Kalman Filter. Prot. Control Mod. Power Syst..

[36] Hur S. (2021). Short-Term Wind Speed Prediction Using Extended Kalman Filter and Machine Learning. Energy Rep..

[37] Bogdanovs N., Belinskis R., Bistrovs V., Petersons E., Ipatovs A. (2021). Forecasting Algorithm Based on Temperature Error Prediction Using Kalman Filter for Management System Development. Latv. J. Phys. Tech. Sci..

[38] Du K.-L., Swamy M.N.S. (2014). Radial Basis Function Networks. Neural Networks and Statistical Learning.

[39] Karamichailidou D., Gerolymatos G., Patrinos P., Sarimveis H., Alexandridis A. (2024). Radial Basis Function Neural Network Training Using Variable Projection and Fuzzy Means. Neural Comput. Appl..

[40] Karamichailidou D., Koletsios S., Alexandridis A. (2022). An RBF Online Learning Scheme for Non-Stationary Environments Based on Fuzzy Means and Givens Rotations. Neurocomputing.

[41] Dey P., Gopal M., Pradhan P., Pal T. (2019). On Robustness of Radial Basis Function Network with Input Perturbation. Neural Comput. Appl..

[42] Que Q., Belkin M. (2020). Back to the Future: Radial Basis Function Network Revisited. IEEE Trans. Pattern Anal. Mach. Intell..

[43] Teng P. (2018). Machine-Learning Quantum Mechanics: Solving Quantum Mechanics Problems Using Radial Basis Function Networks. Phys. Rev. E.

[44] Wu Y., Wang H., Zhang B., Du K.-L. (2012). Using Radial Basis Function Networks for Function Approximation and Classification. ISRN Appl. Math..

[45] Gyamfi K.S., Brusey J., Gaura E. (2022). Differential Radial Basis Function Network for Sequence Modelling. Expert Syst. Appl..

[46] Zainuddin Z., Pauline O. (2008). Function Approximation Using Artificial Neural Networks. WSEAS Trans. Math..

[47] Ferreira A.J.M. (2003). A Formulation of the Multiquadric Radial Basis Function Method for the Analysis of Laminated Composite Plates. Compos. Struct..

[48] Sarra S.A. (2006). Integrated Multiquadric Radial Basis Function Approximation Methods. Comput. Math. Appl..

[49] Kaennakham S., Paewpolsong P., Sriapai N., Tavaen S., Tallón-Ballesteros A.J. (2021). Generalized-Multiquadric Radial Basis Function Neural Networks (RBFNs) with Variable Shape Parameters for Function Recovery. Frontiers in Artificial Intelligence and Applications.

[50] Hefny H.A., Bahnasawi A.A., Abdel Wahab A.H., Shaheen S.I. (1999). Logical Radial Basis Function Networks a Hybrid Intelligent Model for Function Approximation. Adv. Eng. Softw..

[51] Peng H., Ozaki T., Haggan-Ozaki V., Toyoda Y. (2003). A Parameter Optimization Method for Radial Basis Function Type Models. IEEE Trans. Neural Netw..

[52] Mark J. (1996). Introduction to Radial Basis Function Networks. https://cir.nii.ac.jp/crid/1570572699327416064.

[53] Alexandridis A., Sarimveis H., Ninos K. (2011). A Radial Basis Function Network Training Algorithm Using a Non-Symmetric Partition of the Input Space—Application to a Model Predictive Control Configuration. Adv. Eng. Softw..

[54] Arthur D., Vassilvitskii S. (2007). K-Means++: The Advantages of Careful Seeding. Proceedings of the Eighteenth Annual ACM-SIAM Symposium on Discrete Algorithms.

[55] He J., Liu H. The Application of Dynamic K-Means Clustering Algorithm in the Center Selection of RBF Neural Networks. Proceedings of the 2009 Third International Conference on Genetic and Evolutionary Computing.

[56] Liang J., Sarkhel S., Song Z., Yin C., Yin J., Zhuo D. (2022). A Faster k-Means++ Algorithm. arXiv.

[57] Jabbar H.K., Khan R.Z. (2014). Methods to Avoid Over-Fitting and Under-Fitting in Supervised Machine Learning (Comparative Study). Computer Science, Communication and Instrumentation Devices.

[58] Dorugade A.V., Kashid D.N. (2010). Alternative Method for Choosing Ridge Parameter for Regression. Appl. Math. Sci..

